# Research of a Thermodynamic Function ${\left(\frac{\partial p}{\partial x}\right)}_{T,\;x\to 0}$: Temperature Dependence and Relation to Properties at Infinite Dilution

**DOI:** 10.3390/ijms232112998

**Published:** 2022-10-27

**Authors:** Jiahuan Zheng, Xia Chen, Yan Wang, Qichao Sun, Wenting Sun, Lianying Wu, Yangdong Hu, Weitao Zhang

**Affiliations:** 1 College of Chemistry and Chemical Engineering, Ocean University of China, Qingdao 266100, China; 2 Institute of Chemical Engineering, Guangdong Academy of Science, Guangzhou 510665, China; 3 College of Chemistry and Chemical Engineering, Qingdao University, Qingdao 266071, China

**Keywords:** temperature dependence, infinite dilution activity coefficient, solubility, Henry’s law constants, high pressure

## Abstract

In this work, we propose the idea of considering ${\left(\frac{\partial p}{\partial x}\right)}_{T,\;x\to 0}$ as an infinite dilution thermodynamic function. Our research shows that ${\left(\frac{\partial p}{\partial x}\right)}_{T,x\to 0}$ as a thermodynamic function is closely related to temperature, with the relation being simply expressed as: $\ln{\left(\frac{\partial p}{\partial x}\right)}_{T,\;x\to 0}=\frac{A}{T}+B$. Then, we use this equation to correlate the isothermal vapor–liquid equilibrium (VLE) data for 40 systems. The result shows that the total average relative deviation is 0.15%, and the total average absolute deviation is 3.12%. It indicates that the model correlates well with the experimental data. Moreover, we start from the total pressure expression, and use the Gibbs–Duhem equation to re-derive the relationship between ${\left(\frac{\partial p}{\partial x}\right)}_{T,x\to 0}$ and the infinite dilution activity coefficient ($\gamma^{\infty }$) at low pressure. Based on the definition of partial molar volume, an equation for ${\left(\frac{\partial p}{\partial x}\right)}_{T,x\to 0}$ and gas solubility at high pressure is proposed in our work. Then, we use this equation to correlate the literature data on the solubility of nitrogen, hydrogen, methane, and carbon dioxide in water. These systems are reported at temperatures ranging from 273.15 K to 398.15 K and pressures up to 101.325 MPa. The total average relative deviation of the predicted values with respect to the experimental data is 0.08%, and the total average absolute deviation is 2.68%. Compared with the Krichevsky–Kasarnovsky equation, the developed model provides more reliable results.

## 1. Introduction

Knowledge of the phase-equilibrium behavior of solutions at infinite dilution is important not only for phase-equilibrium calculations of a dilute solution, but also for high-concentration solutions. Indeed, the phase-equilibrium properties of the dilute region are widely used in the process design of unit operations such as distillation, extraction, and absorption [1,2,3]. Hence, the study of infinite dilution thermodynamic properties is a crucial topic. There are two functions used to describe thermodynamic properties at infinite dilution, i.e., the infinite dilution activity coefficient ($\gamma^{\infty }$) and Henry’s constant ($H_{i}$). The former can be used to calculate relative volatility, partition coefficients, and phase-equilibrium data [4,5,6,7]. The latter is used to describe the partitioning capacity of a compound in the gas/water phase, mainly calculating the solubility of the gas in liquids [8,9,10].

Several experimental techniques are available to determine $\gamma^{\infty }$, among which the static differential technique and differential ebulliometry are common means. In the static differential method, the pressure difference between two static units of pure solvent and dilute solution is measured in a constant temperature bath. Then, the difference is used to plot the composition-pressure diagram of the liquid phase at equilibrium to obtain ${\left(\frac{\partial p}{\partial x}\right)}_{T,x\to 0}$. However, a differential ebulliometer is used to measure the change in the boiling point of a solvent to obtain ${\left(\frac{\partial T}{\partial x}\right)}_{p,x\to 0}$. In these methods, $\gamma^{\infty }$ is calculated from the relationship derived from Gautreaux and Coates [11]. Earlier studies have indicated that accurate data from these two methods can be obtained if strictly considering the liquid and vapor hold-ups in various parts of the systems [12,13]. Experimental data for Henry’s constant can be calculated using independently measured solubility and partial pressure [10]. Moreover, it can be obtained by the static differential technique. Ayuttaya et al. [14] reported a method for determining $H_{i}$ with a differential static cell. The results suggested that the pressure accuracy influenced the uncertainty of the determined Henry’s constant.

As one of the thermodynamic properties at infinite dilution, the value of $\gamma^{\infty }$ is closely related to temperature. Currently, the theoretical model of $\gamma^{\infty }$ and temperature is derived from the relationship between the activity coefficient and the partial molar excess function, which is expressed as: $\ln\gamma_{i}^{\infty }=\frac{\Delta h_{i}^{E,\infty }}{RT}-\frac{\Delta s_{i}^{E,\infty }}{R}$. Assuming infinite dilution molar entropy ($\Delta h^{E,\infty }$) and infinite dilution molar enthalpy ($\Delta s^{E,\infty }$) are constants within a certain temperature range, the above relationship expresses a two-parameter model [7,15,16]. The subsequent three-parameter model [17], four-parameter model [18], and five-parameter model with the introduction of molecular descriptors *X_i_* [19] were developed from this equation. Notably, although the introduction of *X_i_* improves the correlation accuracy, this equation contains five variables, which complicates the relation between $\gamma^{\infty }$ and temperature.

The pressure effect has a negligible effect on $H_{i}$ when the pressure is low. In this case, $H_{i}$ is closely related to the temperature. Considerable research efforts have been devoted to the two-parameter model [20,21]. The results of the correlation between $H_{i}$ and temperature were not satisfactory for systems with a wide range of temperatures. When considering the change in dissolution heat with temperature, the relationship between $H_{i}$ and temperature was developed into a three-parameter model and a four-parameter model [22]. Although the correlation accuracy of these two models is high over a wide temperature range, the relationship between $H_{i}$ and temperature becomes complicated due to too many parameters, which is not favorable for engineering applications. However, the effect of pressure on $H_{i}$ at high pressures is not negligible. Hence, it is necessary to study $H_{i}$ at high pressures. Thus far, much work has focused on $\gamma^{\infty }$ and $H_{i}$, where both $\gamma^{\infty }$ and $H_{i}$ can be obtained by experimental determination of ${\left(\frac{\partial p}{\partial x}\right)}_{T,x\to 0}$.

In this study, we consider ${\left(\frac{\partial p}{\partial x}\right)}_{T,x\to 0}$ as an infinite dilution thermodynamic function. With this viewpoint, we propose a novel correlation model of ${\left(\frac{\partial p}{\partial x}\right)}_{T,x\to 0}$ with temperature. Based on the calculation results of 40 systems, the novel model is proved to be objective and rational. The outstanding feature of this model is that there are only two adjustable parameters, which can be more easily adopted in engineering applications. Furthermore, as a thermodynamic property, ${\left(\frac{\partial p}{\partial x}\right)}_{T,x\to 0}$ can be used to calculate other thermodynamic properties of infinite dilution, such as $\gamma^{\infty }$ and $H_{i}$. Considering the relationship between ${\left(\frac{\partial p}{\partial x}\right)}_{T,x\to 0}$ and $H_{i}$ at high pressures, a new model is proposed to calculate the solubility of the gas in water. The main feature of this model is that the solubility at a high pressure can be expressed as the property ${\left(\frac{\partial v}{\partial p}\right)}_{T,x\to 0}$ of the pure solvent and the property ${\left(\frac{\partial p}{\partial x}\right)}_{T,x\to 0}$ of the mixture at a low pressure, which constitutes a substantial addition to our understanding of an infinitely diluted solution from a thermodynamic viewpoint.

The rest of this paper is organized as follows. Section 2 gives details of the theory regarding the relationship between ${\left(\frac{\partial p}{\partial x}\right)}_{T,x\to 0}$ and other infinite dilution thermodynamic functions. Section 3 discusses the source of ${\left(\frac{\partial p}{\partial x}\right)}_{T,x\to 0}$. We propose a model to describe the relationship between ${\left(\frac{\partial p}{\partial x}\right)}_{T,x\to 0}$ and temperature. Then, all proposed models are validated using literature data. Finally, our conclusion is stated in Section 4.

## 2. Theory

### 2.1. Definition of ${\left(\frac{\partial p}{\partial x}\right)}_{T,x\to 0}$

For a binary isothermal vapor–liquid equilibrium system, ${\left(\frac{\partial p}{\partial x}\right)}_{T,x\to 0}$ is given by the partial derivative of p with respect to x, where p is the total pressure and x is the molar fraction of solute in the liquid phase. It is notable that x tends to zero. For convenience, the term *x* in the latter is equivalent to $x_{1}$, and the solute is denoted as component “1”, while the solvent is component “2”. It can not only describe the nonideality of the solution but also reflect the interaction between the solute and solvent at infinite dilution.

### 2.2. Relationship between ${\left(\frac{\partial p}{\partial x}\right)}_{T,x\to 0}$ and $\gamma^{\infty }$

For a binary system, the total pressure equation [23] at a low pressure can be described as Equation (1):

(1) $$p=x_{1}\gamma_{1}p_{1}^{s}+x_{2}\gamma_{2}p_{2}^{s}$$

where $p_{1}^{s}$ is the saturated vapor pressure of the solute, $p_{2}^{s}$ is the saturated vapor pressure of the solvent. It is important to emphasize that this equation applies to a low pressure. At a constant temperature, the activity coefficients $\gamma_{1}$ and $\gamma_{2}$ are only a function of the composition. For binary systems, the relationship between the two components is described as:

(2) $$x_{1}+x_{2}=1$$

where $x_{1}$ is the molar fraction of component 1 in the liquid phase, $x_{2}$ is the molar fraction of component 2 in the liquid phase. Therefore, taking the partial derivative of $x_{1}$ at isothermal conditions, we find:

(3) $${\left(\frac{\partial p}{\partial x_{1}}\right)}_{T}={\left(\frac{\partial \gamma_{1}}{\partial x_{1}}\right)}_{T}p_{1}^{s}x_{1}+\gamma_{1}p_{1}^{s}-{\left(\frac{\partial \gamma_{2}}{\partial x_{1}}\right)}_{T}p_{2}^{s}x_{1}-\gamma_{2}p_{2}^{s}+{\left(\frac{\partial \gamma_{2}}{\partial x_{1}}\right)}_{T}p_{2}^{s}$$

According to the expression of the Gibbs–Duhem equation for the binary system:

(4) $$x_{1}\mathrm{dln}\gamma_{1}+x_{2}\mathrm{dln}\gamma_{2}=\frac{h^{E}}{RT^{2}}dT+\frac{V^{E}}{RT}dp$$

where $h^{E}$ is the excess enthalpy, $V^{E}$ is the excess volume, R is the universal gas constant, while the temperature remains a constant, dT=0. The liquids can be considered as an ideal solution at infinite dilution [24,25], $V^{E}=0$. It is convenient to rewrite Equation (4) as:

(5) $$x_{1}\mathrm{dln}\gamma_{1}+x_{2}\mathrm{dln}\gamma_{2}=0$$

Dividing both sides by $dx_{1}$, Equation (5) can be represented as follows:

(6) $$x_{1}\frac{\mathrm{dln}\gamma_{1}}{dx_{1}}+x_{2}\frac{\mathrm{dln}\gamma_{2}}{dx_{1}}=0$$

The above equation is equivalent to:

(7) $$\frac{x_{1}}{\gamma_{1}}\frac{d\gamma_{1}}{dx_{1}}+\frac{x_{2}}{\gamma_{2}}\frac{d\gamma_{2}}{dx_{1}}=0$$

From Equation (7), as $x_{1}$ approaches zero, $\frac{x_{2}}{\gamma_{2}}\frac{d\gamma_{2}}{dx_{1}}\to 0$. Based on the normalized description of the activity coefficient: as the molar fraction of component 2 approaches 1, its activity coefficient converges to 1. Thus, when $x_{1}\to 0$, ${\left(\frac{\partial \gamma_{1}}{\partial x_{1}}\right)}_{T}p_{1}^{s}x_{1}\to 0$, ${\left(\frac{\partial \gamma_{2}}{\partial x_{1}}\right)}_{T}\to 0$, substituting Equation (7) into Equation (3), it becomes:

(8) $${\left(\frac{\partial p}{\partial x_{1}}\right)}_{T,x_{1}\to 0}=\gamma_{1}^{\infty }p_{1}^{s}-p_{2}^{s}$$

Notice that Equation (8) is consistent with the formula reported by Gautreaux and Coates at a low pressure [11]. The difference is that the starting point in our work is the expression of the total pressure at a low pressure. The physical meaning of the derivation in our work is clear and its assumptions are accessible. This is because $\frac{V^{E}}{RT}$ is usually considered as a negligible factor in many studies, and Equation (5) is a common formula that can be used to examine activity coefficient experimental data for thermodynamic consistency [26,27,28].

### 2.3. Relationship between ${\left(\frac{\partial p}{\partial x}\right)}_{T,x\to 0}$ and $H_{i}$

Henry’s law is usually described as a limit law, that is:

(9) $$H_{1}=\underset{x_{1}\to 0}{\lim}\frac{f_{1}^{L}}{x_{1}}$$

When considering an ideal solution based on Raoult’s law to define the activity coefficient, the liquid phase fugacity can be expressed as:

(10) $$f_{1}^{L}=x_{1}\gamma_{1}f_{1}$$

where $f_{1}$ is the fugacity of the pure liquid at solution temperature and pressure. Thus, combining Equations (9) and (10), that is:

(11) $$H_{1}=\underset{x_{1}\to 0}{\lim}\gamma_{1}f_{1}$$

At low pressures, $f_{1}$ is replaced by the saturated vapor pressure of the liquid $p_{1}^{s}$. Hence, Equation (11) becomes [7,29]:

(12) $$H_{1}=\gamma_{1}^{\infty }p_{1}^{s}$$

Thus, the expression of the relationship between ${\left(\frac{\partial p}{\partial x}\right)}_{T,x\to 0}$ and $H_{1}$ is obtained directly from Equations (8) and (12):

(13) $${\left(\frac{\partial p}{\partial x_{1}}\right)}_{T,x_{1}\to 0}=H_{1}-p_{2}^{s}$$

Obviously, Equation (13) indicates that there is a simple relationship between ${\left(\frac{\partial p}{\partial x}\right)}_{T,x\to 0}$ and $H_{1}$. In particular, when the solvent saturation vapor pressure $p_{2}^{s}$ is low, Equation (10) becomes:

(14) $${\left(\frac{\partial p}{\partial x_{1}}\right)}_{T,x_{1}\to 0}=H_{1}$$

This reveals that ${\left(\frac{\partial p}{\partial x}\right)}_{T,x\to 0}$ is Henry’s coefficient in some special conditions. Therefore, it is appropriate to consider it as a thermodynamic property alone.

### 2.4. Relationship between ${\left(\frac{\partial p}{\partial x}\right)}_{T,x\to 0}$ and Solubility at High Pressures

The above consideration represents the case of a low pressure. However, the influence of the pressure effect on Henry’s constant cannot be ignored when the pressure is high. Therefore, it is necessary to extend the expressions of ${\left(\frac{\partial p}{\partial x_{1}}\right)}_{T,x_{1}\to 0}$ and $H_{1}$ under high pressures.

From strict thermodynamic relations, we can obtain:

(15) $${\left(\frac{\partial \ln f_{1}^{L}}{\partial p}\right)}_{T,x}=\frac{{\bar{v}}_{1}}{RT}$$

where ${\bar{v}}_{1}$ is the partial molar volume of the solute in liquids. Substituting this result into Equation (9), we get:

(16) $${\left(\frac{\partial \ln H_{1}}{\partial p}\right)}_{T}=\frac{{\bar{v}}_{1}^{\infty }}{RT}$$

where ${\bar{v}}_{1}^{\infty }$ is the partial molar volume of the solute at infinite dilution. Assuming that $f_{1}$ is proportional to $x_{1}$ at constant temperature and pressure, a more general form of Henry’s law can be obtained:

(17) $$\ln\frac{f_{1}}{x_{1}}=\ln H_{1}^{\left(p^{0}\right)}+\frac{\int_{p^{0}}^{p}{\bar{v}}_{1}^{\infty }dp}{RT}$$

where $p^{0}$ is the reference pressure, $p^{0}$ = 101.325 kPa, $H_{1}^{\left(p^{0}\right)}$ is the Henry’s constant at 101.325 kPa. By definition, ${\bar{v}}_{1}$ can be written as:

(18) $${\bar{v}}_{1}={\left(\frac{\partial p}{\partial V}\right)}_{T,p,n_{2}}$$

Thus, the partial molar volume, as defined by Equation (18), can be evaluated using the following transformation:

(19) $${\bar{v}}_{1}=-\frac{{\left(\partial p/\partial n_{1}\right)}_{T,V,n_{2}}}{{\left(\partial p/\partial V\right)}_{T,n_{1},n_{2}}}$$

where ${\left(\frac{\partial p}{\partial V}\right)}_{T,n_{1},n_{2}}$ describes the properties of solvents, which can be obtained by the equation of state. Since V changes near the saturated volume at infinite dilution, ${\left(\frac{\partial p}{\partial n_{1}}\right)}_{T,V,n_{2}}$ can be written as ${\left(\frac{\partial p}{\partial n_{1}}\right)}_{T,x_{1}\to 0}$. Thus:

(20) $${\left(\frac{\partial p}{\partial n_{1}}\right)}_{T,x_{1}\to 0}={\left(\frac{\partial p}{\partial x_{1}}\right)}_{T,x_{1}\to 0}{\left(\frac{\partial x_{1}}{\partial n_{1}}\right)}_{T,x_{1}\to 0}$$

Substituting Equation (19) to Equation (20) gives:

(21) $${\bar{v}}_{1}^{\infty }=-{\left(\frac{\partial p}{\partial x_{1}}\right)}_{T,x_{1}\to 0}{\left(\frac{\partial v}{\partial p}\right)}_{T,x_{1}\to 0}$$

where ${\left(\frac{\partial p}{\partial x_{1}}\right)}_{T,x_{1}\to 0}$ represents the properties of the solute at a low pressure. Generally, it is difficult to determine ${\bar{v}}_{1}^{\infty }$ by experiment. In this work, ${\bar{v}}_{1}^{\infty }$ is expressed as the properties of the solvent at a high pressure and the solute at a low pressure, which provides a new idea for obtaining ${\bar{v}}_{1}^{\infty }$.

Hence, an equation for ${\left(\frac{\partial p}{\partial x}\right)}_{T,x\to 0}$ and solubility at high pressures can be written as:

(22) $$\ln\frac{f_{1}}{x_{1}}=\ln H_{1}^{\left(p^{0}\right)}-\frac{1}{RT}{\left(\frac{\partial p}{\partial x_{1}}\right)}_{T,x_{1}\to 0}\int_{p^{0}}^{p}{\left(\frac{\partial v}{\partial p}\right)}_{T,x_{1}\to 0}dp$$

Considering the variation in the solute activity coefficient with molar composition, we added the activity coefficient for correction. Therefore, the final equation can be written as follows:

(23) $$\ln\frac{f_{1}}{x_{1}}=\ln H_{1}^{\left(p^{0}\right)}-\frac{1}{RT}{\left(\frac{\partial p}{\partial x_{1}}\right)}_{T,x_{1}\to 0}\int_{p^{0}}^{p}{\left(\frac{\partial v}{\partial p}\right)}_{T,x_{1}\to 0}dp+\ln\gamma_{1}$$

### 2.5. Relationship between ${\left(\frac{\partial p}{\partial x}\right)}_{T,x\to 0}$ and ${\left(\frac{\partial T}{\partial x}\right)}_{p,x\to 0}$

Differential ebulliometry [30] uses ${\left(\frac{\partial T}{\partial x}\right)}_{p,x\to 0}$, while the differential statics method [31] uses ${\left(\frac{\partial p}{\partial x}\right)}_{T,x\to 0}$ to calculate $\gamma^{\infty }$. Similarly, regarding the properties of an infinitely diluted solution, there should be a certain relationship between ${\left(\frac{\partial T}{\partial x}\right)}_{p,x\to 0}$ and ${\left(\frac{\partial p}{\partial x}\right)}_{T,x\to 0}$.

According to the Gibbs phase rule, pressure is a function of temperature and composition for a binary system:

(24) p(T,x)=0

(25) $$dp={\left(\frac{\partial p}{\partial T}\right)}_{x}dT+{\left(\frac{\partial p}{\partial x}\right)}_{T}dx$$

When dp is equal to zero at a constant pressure, we get:

(26) $${\left(\frac{\partial p}{\partial T}\right)}_{x}{\left(dT\right)}_{p}+{\left(\frac{\partial p}{\partial x}\right)}_{T}{\left(dx\right)}_{p}=0$$

Notably, ${\left(\frac{\partial p}{\partial T}\right)}_{x}$ converges to $\frac{dp_{2}^{s}}{dT}$ as *x* approaches zero. Hence, Equation (26) yields the desired result:

(27) $${\left(\frac{\partial p}{\partial x}\right)}_{T,x\to 0}=-{\left(\frac{dp_{2}^{s}}{dT}\right)}_{x\to 0}{\left(\frac{\partial T}{\partial x}\right)}_{p,x\to 0}$$

Equation (27) is consistent with the descriptions of Van Ness and Abbott [32], which reflects the strict relationship between ${\left(\frac{\partial p}{\partial x}\right)}_{T,x\to 0}$ and ${\left(\frac{\partial T}{\partial x}\right)}_{p,x\to 0}$. Several additional points warrant explanation. In general, ${\left(\frac{dp_{2}^{s}}{dT}\right)}_{x\to 0}>0$, ${\left(\frac{\partial p}{\partial x}\right)}_{T,x\to 0}$, and ${\left(\frac{\partial T}{\partial x}\right)}_{p,x\to 0}$ have opposite signs. The available experimental data show that ${\left(\frac{\partial T}{\partial x}\right)}_{p,x\to 0}<0$; thus, ${\left(\frac{\partial p}{\partial x}\right)}_{T,x\to 0}$ should be positive. In addition, it is clear from Equation (27) that ${\left(\frac{\partial p}{\partial x}\right)}_{T,x\to 0}$ and ${\left(\frac{\partial T}{\partial x}\right)}_{p,x\to 0}$ can be extrapolated from each other if ${\left(\frac{dp_{2}^{s}}{dT}\right)}_{x\to 0}$ is accurate. Generally, ${\left(\frac{\partial T}{\partial x}\right)}_{p,x\to 0}$ is relatively easy to measure [30], and ${\left(\frac{\partial p}{\partial x}\right)}_{T,x\to 0}$ can be obtained by determining ${\left(\frac{\partial T}{\partial x}\right)}_{p,x\to 0}$ using Equation (27). Therefore, as an important thermodynamic property, ${\left(\frac{\partial p}{\partial x}\right)}_{T,x\to 0}$ can be measured not only directly but also indirectly by ${\left(\frac{\partial T}{\partial x}\right)}_{p,x\to 0}$.

## 3. Results and Discussion

### 3.1. Source of ${\left(\frac{\partial p}{\partial x}\right)}_{T,x\to 0}$

**Figure 1:**
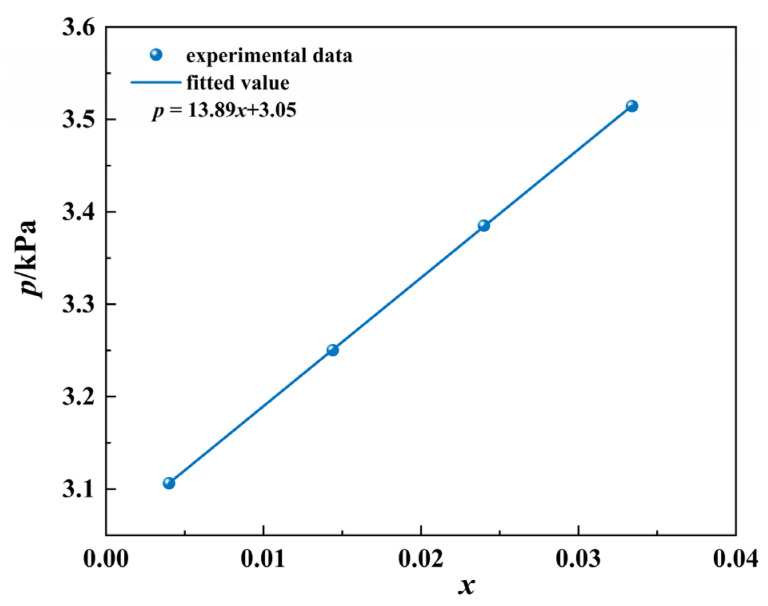
Relationship between total pressure and liquid phase composition *x* of water in a tert-amyl alcohol system at 303.32 K.

We select the isothermal vapor–liquid equilibrium (VLE) data [33,34,35,36,37] with the pressure as a function of the molar fraction for regression at low concentration ranges ($x_{1}<0.05$), typically 3–7 points. As shown in Figure 1, the VLE data of water in the tert-amyl alcohol system are regressed using the least squares method, where x represents the molar fraction of water in tert-amyl alcohol. It was found that p is linearly related to x, which can be written as Equation (28). More interestingly, Equation (28) can also be applied to other systems. Thus, the slope a is ${\left(\frac{\partial p}{\partial x}\right)}_{T,x\to 0}$ at a constant temperature.

(28) p=ax+b

### 3.2. Correlation of ${\left(\frac{\partial p}{\partial x}\right)}_{T,x\to 0}$ with Temperature

As previously noted, there is a simple relationship between ${\left(\frac{\partial p}{\partial x}\right)}_{T,x\to 0}$ and $H_{i}$ in Equations (13) and (14). In particular, when $p_{2}^{s}$ is very low, ${\left(\frac{\partial p}{\partial x}\right)}_{T,x\to 0}$ is equal to $H_{i}$.

Several excellent studies which describe the relationship between $H_{i}$ and temperature are all two-parameter models [7,20,38], with the relations being expressed as follows:

(29) $$\ln H_{1}=\frac{c}{T}+d$$

Therefore, we propose a relationship between ${\left(\frac{\partial p}{\partial x}\right)}_{T,x\to 0}$ and temperature which can be written as follows:

(30) $$\ln{\left(\frac{\partial p}{\partial x}\right)}_{T,\;\;x\to 0}=\frac{A}{T}+B$$

**Figure 2:**
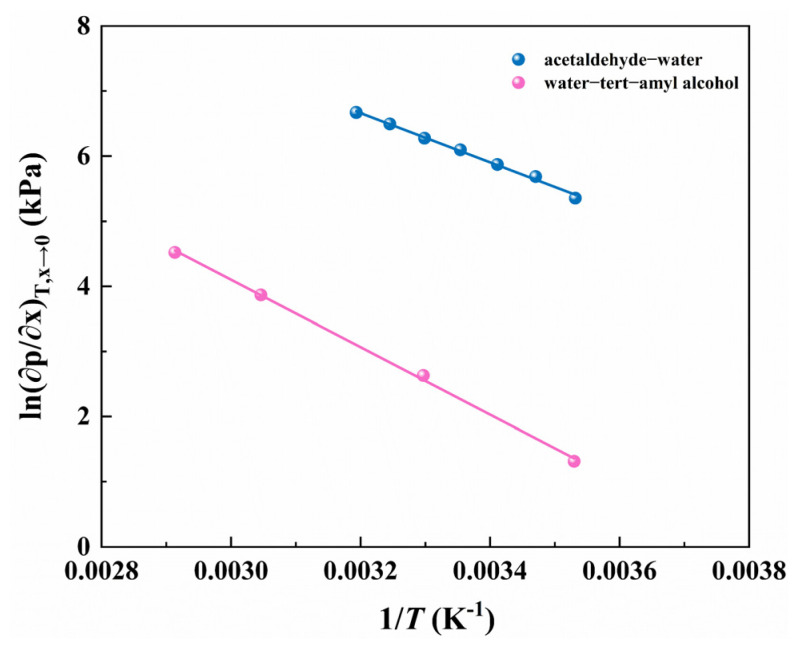
Relationship between ${\left(\frac{\partial p}{\partial x}\right)}_{T,x\to 0}$ and temperature in different systems.

To prove the correctness of Equation (29), acetaldehyde–water and water–tert-amyl-alcohol systems are correlated by this relationship, as illustrated in Figure 2. The results show that the correlation coefficient $R^{2}>0.99$, which indicates a good linear relationship between $\ln{\left(\frac{\partial p}{\partial x}\right)}_{T,\;x\to 0}$ and the inverse of temperature.

Forty groups of isothermal VLE data containing 156 values of ${\left(\frac{\partial p}{\partial x}\right)}_{T,x\to 0}$ are used to test the correlation model in this work, including 20 groups of organic–aqueous systems, 5 groups of aqueous–organic systems, and 15 groups of organic–organic systems. The correlation of ${\left(\frac{\partial p}{\partial x}\right)}_{T,x\to 0}$ and temperature is performed using Equation (30). The average deviations of model parameters and experimental data of the correlation result equation are listed in Table 1. We use two deviations to obtain conclusions about the accuracy of the given model [39], where the average absolute deviation (A.A.D. (%)) is calculated based on Equation (31) and the average relative deviation (A.R.D. (%)) is calculated based on Equation (32).

(31) $$A.A.D.\left(\%\right)=\frac{100}{N_{p}}\overset{N_{p}}{\underset{i=1}{\sum }}{\left[\frac{\left|{\left(\frac{\partial p}{\partial x}\right)}_{T,\;\;x\to 0,\exp.}-{\left(\frac{\partial p}{\partial x}\right)}_{T,\;\;x\to 0,\mathrm{cal}.}\right|}{{\left(\frac{\partial p}{\partial x}\right)}_{T,\;\;x\to 0,\exp.}}\right]}_{i}$$

(32) $$A.R.D.\left(\%\right)=\frac{100}{N_{p}}\overset{N_{p}}{\underset{i=1}{\sum }}{\left[\frac{{\left(\frac{\partial p}{\partial x}\right)}_{T,\;\;x\to 0,\exp.}-{\left(\frac{\partial p}{\partial x}\right)}_{T,\;\;x\to 0,\mathrm{cal}.}}{{\left(\frac{\partial p}{\partial x}\right)}_{T,\;\;x\to 0,\exp.}}\right]}_{i}$$

Satisfactory results are obtained from the correlation of aqueous solution systems of 2-butanone and ethyl acrylate, and the values of A.A.D. (%) for these systems are less than 1%. The calculation results of the model are accurate for the correlation of small molecule solution systems, especially for aqueous solutions of organic compounds. However, the largest deviation is found for the methyl-acetate–ethanol, with an A.A.D. (%) of 6.69%. Such deviation occurs because the mole fraction of methyl acetate in ethanol is close to 0.05 and the solution concentration is high. Hence, a more accurate value of ${\left(\frac{\partial p}{\partial x}\right)}_{T,x\to 0}$ can be obtained as x approaches 0, when the correlation between ${\left(\frac{\partial p}{\partial x}\right)}_{T,x\to 0}$ and temperature improves.

### 3.3. Calculate ${\left(\frac{\partial p}{\partial x}\right)}_{T,x\to 0}$ from ${\left(\frac{\partial T}{\partial x}\right)}_{p,x\to 0}$

In addition to the correlation model, we can also determine ${\left(\frac{\partial T}{\partial x}\right)}_{p,x\to 0}$ by using ${\left(\frac{\partial p}{\partial x}\right)}_{T,x\to 0}$. The isobaric VLE data [33,34,35] of eight systems are selected to calculate their ${\left(\frac{\partial T}{\partial x}\right)}_{p,x\to 0}$ at different temperature and pressure in this work, and ${\left(\frac{\partial p}{\partial x}\right)}_{T,x\to 0}$ is calculated from Equation (27), where $p^{s}$ is calculated by the Antoine equation with parameters from the NIST database (https://webbook.nist.gov/, accessed on 28 March 2020). The results indicate that the calculation values are in good agreement with the values of ${\left(\frac{\partial p}{\partial x}\right)}_{T,x\to 0}$ obtained from isothermal VLE data, as shown in Table 2. It is noteworthy that for systems with low relative volatilities [40], ${\left(\frac{\partial p}{\partial x}\right)}_{T,x\to 0}$ is not easy to determine, but ${\left(\frac{\partial T}{\partial x}\right)}_{p,x\to 0}$ can be well measured by differential ebulliometry. In this case, an accurate value of ${\left(\frac{\partial p}{\partial x}\right)}_{T,x\to 0}$ can be calculated from ${\left(\frac{\partial T}{\partial x}\right)}_{p,x\to 0}$.

### 3.4. Use ${\left(\frac{\partial p}{\partial x}\right)}_{T,x\to 0}$ to Calculate Other Infinite Dilution Properties

As a thermodynamic property, the value of ${\left(\frac{\partial p}{\partial x}\right)}_{T,x\to 0}$ can be calculated from $\gamma^{\infty }$ and $H_{i}$ as shown in Equations (8) and (13). In this section, these two infinite dilution thermodynamic functions are calculated by ${\left(\frac{\partial p}{\partial x}\right)}_{T,x\to 0}$ from isothermal VLE data. Moreover, the accuracy of Equations (22) and (23) are verified using high-pressure solubility data for different systems.

#### 3.4.1. Using ${\left(\frac{\partial p}{\partial x}\right)}_{T,x\to 0}$ to Calculate $\gamma^{\infty }$

We use isothermal VLE data [33,34,35] with the pressure as a linear function of the molar fraction to calculate ${\left(\frac{\partial p}{\partial x}\right)}_{T,x\to 0}$, and then obtain $\gamma^{\infty }$ from Equation (8). The results are shown in Table 3. According to the results of the calculations, most systems are well adapted to the literature, while a few systems such as nitromethane–water show significant differences. This result may be related to the accuracy of the $\gamma^{\infty }$ experimental value; many researchers reported that $\gamma^{\infty }$ measured by different methods showed significant differences [18,41]. Moreover, Sherman et al. [42] evaluated the database of $\gamma^{\infty }$ for non-electrolytes in water and found that the accuracy of the database for measured values is estimated at 10% for $\gamma^{\infty }<1000$.

#### 3.4.2. Using ${\left(\frac{\partial p}{\partial x}\right)}_{T,x\to 0}$ to Calculate $H_{i}$

We calculate ${\left(\frac{\partial p}{\partial x}\right)}_{T,x\to 0}$ from the isothermal VLE data [33,34,35] with the pressure as a linear function of the molar fraction, and we use Equation (13) to obtain $H_{i}$. The calculation results are shown in Table 4. As shown, the calculated results are in good agreement with the literature data, which illustrates the accuracy of the relationship between ${\left(\frac{\partial p}{\partial x}\right)}_{T,x\to 0}$ and $H_{i}$. It can be seen that the saturation vapor pressure of water at 288.15 K is very low, close to zero. Meanwhile, the value of Henry’s constant for acetaldehyde in water is approximately equal to ${\left(\frac{\partial p}{\partial x}\right)}_{T,x\to 0}$. This proves that ${\left(\frac{\partial p}{\partial x}\right)}_{T,x\to 0}$ is reasonable as a thermodynamic property.

### 3.5. Use ${\left(\frac{\partial p}{\partial x}\right)}_{T,x\to 0}$ to Calculate the Solubility of the Gas at High Pressure

As a traditional model, the Krichevsky–Kasarnovsky (K–K) equation is often used to express the effect of high pressures on gas solubility [46], given by:

(33) $$\ln\frac{f_{1}}{x_{1}}=\ln H_{1}^{\left(p^{0}\right)}+\frac{{\bar{v}}_{1}^{\infty }\left(p-p^{0}\right)}{RT}$$

where $p^{0}$ is the reference pressure, and $H_{1}^{\left(p^{0}\right)}$ is Henry’s constant at $p^{0}$.

**Figure 3:**
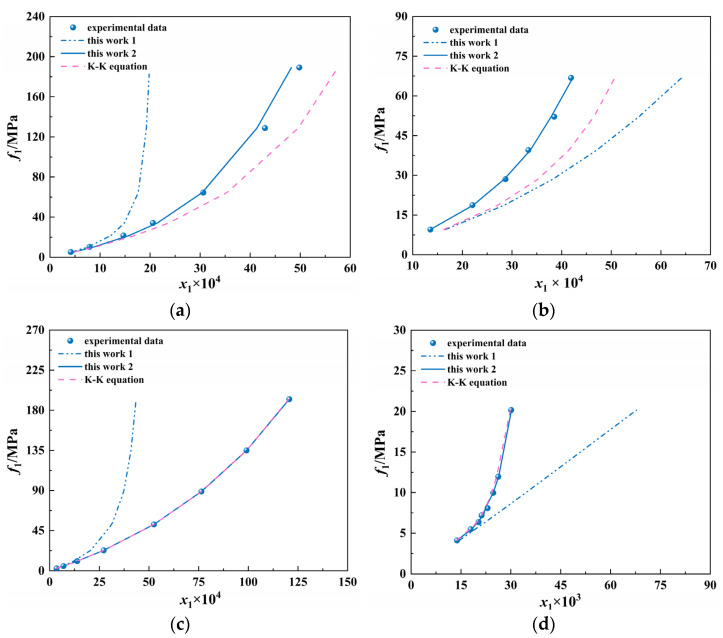
Solubility versus fugacity calculated by different models for nitrogen and methane systems. (**a**) nitrogen, 373.15 K; (**b**) methane, 375.65 K; (**c**) hydrogen, 298.15 K; (**d**) carbon dioxide, 323.15 K; this work 1: calculated from Equation (22); this work 2: calculated from Equation (23).

In this work, we deduce formulas for solubility and ${\left(\frac{\partial p}{\partial x}\right)}_{T,x\to 0}$ at high pressure, as shown in Equations (22) and (23). In both formulae, ${\left(\frac{\partial v}{\partial p}\right)}_{T}$ is the property of pure water, which can be calculated by the equations of IAPWS-IF97 [47]. In Figure 3, we compared the relationship between solubility and fugacity using different models for four aqueous systems. The blue solid lines indicate the best fits to the experimental data (“this work 2”), while pink dotted lines represent K–K equation results. It can be seen that both the proposed model and the K–K equation can describe the solubility of hydrogen and carbon dioxide well in water at high pressures. In particular, the model in this work is superior to the K–K equation for the nitrogen and methane systems. Moreover, Figure 3 shows that the model with the activity coefficient in this work, as illustrated Equation (23), can better describe the effect of high pressures on solubility, especially for systems with pressures up to 101.325 MPa. This suggests that the solubility of gas in water increases at high pressures, and the activity coefficient of the solute in water cannot be ignored.

In addition, four typical systems are selected for comparison with the proposed model, with temperatures ranging from 273.15 K to 398.15 K and pressures up to 101.325 MPa. The average deviations of the calculated results concerning experimental data in this work are listed in Table S1.

The results show that the A.A.D. (%) of the model with the activity coefficient for 13 groups is as low as 2.68%, and the model prediction of this paper is more accurate compared with that of the K–K equation. In this study, we find that the activity coefficient of the solute is closely related to the composition. The parameters between the activity coefficient and the composition are shown in Table 5. It is worth noting that for hydrogen and nitrogen systems, the relationship between the activity coefficient and the composition can be simply expressed as a linear relationship. However, for systems with relatively strong interactions with water, such as carbon dioxide and methane systems, it can be expressed as a quadratic functional relationship.

.

## 4. Conclusions

In this paper, we propose the idea of considering ${\left(\frac{\partial p}{\partial x}\right)}_{T,x\to 0}$ as a thermodynamic function, which reflects the interaction of the solute and solvent in the dilute region. Based on this idea, a model is proposed to describe the relationship between ${\left(\frac{\partial p}{\partial x}\right)}_{T,x\to 0}$ and temperature in our work. We find that $\ln{\left(\frac{\partial p}{\partial x}\right)}_{T,x\to 0}$ is linearly related to the reciprocal of temperature. The values of parameters *A* and *B* are reported based on isothermal VLE data from 40 groups of small-molecule systems. The results show that the total A.R.D. (%) of the calculated results compared with the experimental data is 0.15%. This suggests that the proposed model matches the experimental data well.

In addition, we re-derive the relationship between ${\left(\frac{\partial p}{\partial x}\right)}_{T,x\to 0}$ and $\gamma^{\infty }$ at low pressures, in terms of the Gibbs–Duhem equation. We also describe the relationship between ${\left(\frac{\partial p}{\partial x}\right)}_{T,x\to 0}$ and other infinite dilution thermodynamic functions. Based on these relationships, we can obtain the value of ${\left(\frac{\partial p}{\partial x}\right)}_{T,x\to 0}$ from $\gamma^{\infty }$ or $H_{i}$ data, or by using isothermal VLE data and differential ebulliometry. Furthermore, we put forward a new solubility model considering the relationship between ${\left(\frac{\partial p}{\partial x}\right)}_{T,x\to 0}$ and $H_{i}$ at high pressures. The parameters of the proposed model are determined according to the experimental data of four systems. Comparing the calculated results with the experimental data, the A.A.D. (%) of the proposed model is 2.68%, and the A.R.D. (%) of the proposed model is only 0.08%. Compared to the classical K–K model, the new model can correlate the solubility data more accurately. Overall, these results give a comprehensive understanding of infinite dilution properties, which can facilitate improvements in promising applications for chemical process design.

## Figures and Tables

**Table 1:** The values of the parameters and calculated results of the proposed model.

Solute	Solvent	Temperature Range	*N*_p_	Parameters		A.A.D. (%)	A.R.D. (%)
		K		*A*	*B*		
Methanol	Water	[298.15, 343.15]	5	−4739.83	14.42	3.84	0.11
2-Propanol	Water	[298.15, 353.15]	3	−6309.05	20.16	1.26	0.01
Tert-butyl alcohol	Water	[283.15, 348.15]	7	−3771.91	12.25	5.19	0.19
2-Pentanol	Water	[343.15, 363.15]	3	−5310.75	18.02	3.08	0.05
Phenol	Water	[323.15, 363.14]	5	−5437.36	14.06	5.85	0.22
2-Butanone	Water	[323.15, 343.15]	3	−2308.43	9.22	0.10	0.00
Acrolein	Water	[273.15, 326.55]	4	−3539.70	13.57	5.99	0.26
3-Methylpyridine	Water	[308.15, 338.15]	4	−3025.80	11.90	2.34	0.03
2-Ethylbutylamine	Water	[298.15, 313.15]	3	−6884.93	24.26	0.83	0.00
*N, N*-Dimethyl-tert-butylamine	Water	[293.15, 318.05]	5	−6096.80	21.32	3.44	0.08
Ethyl acetate	Water	[298.15, 343.15]	5	−4983.99	18.40	5.50	−0.07
Ethyl acrylate	Water	[333.20, 353.20]	3	−1793.23	9.67	0.52	0.00
Tert-Butyl ethyl ether	Water	[293.15, 313.15]	3	−3979.73	17.36	1.55	1.54
Nitromethane	Water	[294.15, 323.15]	4	−3576.85	12.24	3.25	1.10
*N, N*-Diethyl isopropyl amine	Water	[283.15, 313.15]	4	−6750.68	23.21	5.51	0.17
Diethylamine	Water	[311.50, 329.95]	3	−3238.10	10.05	3.53	0.07
Oxirane	Water	[283.15, 298.15]	3	−3813.18	15.14	0.92	0.00
Acetaldehyde	Water	[283.15, 313.15]	7	−3791.90	14.18	2.35	0.04
Butyraldehyde	Water	[323.15, 353.15]	5	−4643.35	17.08	3.26	0.06
1,4-Dioxane	Water	[293.15, 343.15]	5	−4080.83	11.84	6.37	0.25
Water	Propylene carbonate	[288.15, 300.15]	3	−11,493.77	37.21	3.23	0.07
Water	2-Butoxyethanol	[358.15, 371.19]	4	−6324.31	17.98	2.30	0.02
Water	2-Pentanol	[343.15, 363.15]	3	−4480.93	13.07	1.67	0.01
Water	2-Methyl-2-butanol	[283.3, 343.25]	4	−5180.75	15.03	3.62	0.09
Water	Furfural	[310.95, 366.45]	3	−3330.47	10.10	3.09	0.05
Methanol	Tetrachloromethane	[303.15, 353.15]	6	−3588.41	13.22	5.22	0.17
Methanol	Benzene	[293.15, 318.15]	3	−2310.70	8.55	5.54	0.18
Methanol	Hexanes	[308.15, 348.15]	4	−4626.61	16.23	2.54	0.04
Tetrachloromethane	Ethanol	[293.15, 338.15]	3	−3349.49	10.68	1.00	0.01
Chloroform	Ethanol	[308.15, 328.15]	5	−3882.67	11.95	1.71	0.02
Acetone	Ethanol	[305.15, 321.15]	3	−2775.27	8.69	1.70	0.01
Methyl acetate	Ethanol	[323.15, 353.15]	4	−4557.57	14.01	6.69	0.23
Ethyl acetate	Ethanol	[313.15, 343.15]	3	−3634.74	10.77	4.28	0.10
Ethyl acetate	2-Methoxyethanol	[343.15, 363.15]	3	−4390.60	12.83	4.56	0.16
Benzene	1-Propanol	[308.18, 338.15]	5	−4067.32	12.52	1.54	0.02
Benzene	Isopropanol	[303.15, 333.15]	3	−6344.98	19.28	0.07	−0.06
*N*-methylaniline	Ethylene glycol	[368.15, 418.15]	3	−4732.63	11.12	3.08	0.06
2-Butanone	2-Methoxyethanol	[343.15, 363.15]	3	−5783.97	16.80	1.27	0.01
Iso-butanol	Dimethyl sulfoxide	[353.15, 378.15]	3	−4779.21	12.09	6.48	0.24
Toluene	Pentanol	[303.15, 383.15]	5	−3621.20	10.10	4.14	0.09
Average						3.21	0.15

**Table 2:** Calculate the value of ${\left(\frac{\partial p}{\partial x}\right)}_{T,x\to 0}$ from ${\left(\frac{\partial T}{\partial x}\right)}_{p,x\to 0}$.

Solute	Solvent	*T*	*p*	${\left(\frac{\partial T}{\partial x}\right)}_{p,\;x\to 0}$	${\left(\frac{dp^{s}}{dT}\right)}_{x\to 0}$	${\left(\frac{\partial p}{\partial x}\right)}_{T,\;x\to 0,\mathrm{cal}.}$	${\left(\frac{\partial p}{\partial x}\right)}_{T,\;x\to 0,\mathrm{ref}.}$	Ref
		K	kPa	K	kPa/K	kPa	kPa	
Methanol	Water	372.88	101.33	−156.00	4.05	560.33	563.37	[33]
Ethanol	Water	373.13	101.33	−300.63	4.05	1088.23	1113.56	[34]
1-Propanol	Water	323.59	13.17	−244.11	1.01	151.99	149.96	[33]
2-Propanol	Water	342.33	30.40	−443.33	1.01	580.59	574.51	[33]
Acetone	Water	330.34	20.27	−510.34	1.01	421.51	421.51	[34]
Acetone	Water	356.13	59.78	−492.31	2.03	1043.65	1035.54	[33]
Acetone	Water	363.71	80.05	−480.95	3.04	1304.05	1287.84	[33]
Acetone	Water	373.13	101.33	−454.40	4.05	1643.49	1668.82	[33]
Methanol	1,4-Dioxane	374.20	101.33	−394.55	3.04	1220.97	1235.15	[35]
Methanol	Benzene	353.13	101.33	−239.87	3.04	748.79	756.90	[35]
Methanol	Benzene	327.47	45.60	−288.51	2.03	463.06	452.92	[35]
Methanol	Benzene	340.58	69.91	−261.89	2.03	592.75	594.78	[35]
Benzene	1-Propanol	370.35	101.33	−123.68	4.05	476.23	470.15	[35]

**Table 3:** Calculation of $\gamma^{\infty }$ at different temperature by using ${\left(\frac{\partial p}{\partial x}\right)}_{T,x\to 0}$.

Solute	Solvent	*T*	${\left(\frac{\partial p}{\partial x}\right)}_{T,\;x\to 0,\mathrm{VLE}}$	$\gamma_{\mathrm{VLE}}^{\infty }{\;}^{a}$	${\left(\frac{\partial p}{\partial x}\right)}_{T,\;x\to 0,\mathrm{cor}.}$	$\gamma_{\mathrm{cor}.}^{\infty }{\;}^{b}$	$\gamma_{\mathrm{ref}.}^{\infty }$	Ref
		K	atm		atm			
Methanol	Water	323.15	0.77	1.63	0.76	1.61	1.64	[18]
Ethanol	Water	298.28	0.36	5.04	0.35	4.94	5.22	[18]
Tert-butyl alcohol	Water	298.15	0.63	12.16	0.65	12.41	11.91	[43]
Nitromethane	Water	294.15	1.12	30.34	1.08	29.25	34.80	[43]
Nitromethane	Water	296.15	1.12	27.34	1.16	28.23	33.21	[43]
Nitromethane	Water	323.15	3.17	27.38	3.21	27.76	29.10	[43]
Butyraldehyde	Water	343.15	33.93	39.93	34.43	40.51	39.25	[43]
Tetrachloromethane	Ethanol	293.15	0.47	4.43	0.49	4.58	4.90	[43]
Tetrachloromethane	Ethanol	338.15	2.15	3.27	2.24	3.38	3.73	[43]
Chloroform	Ethanol	308.15	0.51	1.62	0.53	1.64	1.67	[43]
Chloroform	Ethanol	318.15	0.77	1.76	0.77	1.75	1.70	[43]
Chloroform	Ethanol	328.15	1.09	1.80	1.13	1.84	1.55	[43]
Acetone	Ethanol	305.15	0.67	1.94	0.67	1.94	2.28	[43]
Acetone	Ethanol	313.15	0.82	1.78	0.85	1.83	2.21	[43]
Acetone	Ethanol	321.15	1.06	1.76	1.06	1.76	2.14	[43]
Ethyl acetate	Ethanol	313.15	0.45	2.50	0.43	2.44	2.77	[43]

^a^ Calculation of $\gamma^{\infty }$ from ${\left(\frac{\partial p}{\partial x}\right)}_{T,x\to 0}$ of gas–liquid equilibrium data. **^b^** Calculation of $\gamma^{\infty }$ from ${\left(\frac{\partial p}{\partial x}\right)}_{T,x\to 0}$ of the correlation model.

**Table 4:** Calculation of $H_{i}$ at different temperatures using ${\left(\frac{\partial p}{\partial x}\right)}_{T,x\to 0}$.

Solute	Solvent	*T*	$p_{2}^{s}$	${\left(\frac{\partial p}{\partial x}\right)}_{T,\;x\to 0,\mathrm{VLE}}$	$H_{1,\mathrm{VLE}}$	${\left(\frac{\partial p}{\partial x}\right)}_{T,\;\;x\to 0,\mathrm{cor}.}$	$H_{1,\mathrm{cor}.}$	$H_{1,\mathrm{ref}.}$	Ref
		K	kPa	kPa	kPa	kPa	kPa	kPa	
Methanol	Water	323.15	12.30	78.08	90.38	77.06	89.36	81.41	[33,44]
Methanol	Water	338.15	25.43	152.76	178.19	153.86	179.29	177.56	[34,44]
Ethanol	Water	298.15	3.23	36.71	39.94	35.89	39.11	41.32	[33,44]
Acetone	Water	298.15	3.06	134.36	137.42	128.60	131.66	141.68	[33,45]
Acetone	Water	303.15	4.12	168.80	172.92	161.21	165.33	172.70	[34,45]
Acetaldehyde	Water	288.15	0.71	294.54	295.25	290.08	290.79	288.29	[33,45]
Acetaldehyde	Water	293.15	2.98	355.17	358.15	369.99	372.97	364.43	[33,45]
Oxirane	Water	293.15	2.33	865.54	867.87	860.94	863.27	-	[33]
Methyl acetate	Water	308.15	5.61	753.33	758.94	748.58	754.19	-	[33]
Tert-butyl ethyl ether	Water	293.41	2.92	4499.67	4502.59	4481.38	4484.30	-	[33]
Tert-butyl ethyl ether	Water	313.15	7.38	10,580.55	10,587.93	10,534.68	10,542.06	-	[33]
Dimethyl isopropyl amine	Water	293.15	2.67	203.96	206.63	202.97	205.64	-	[33]
Water	2-Pentanol	343.15	12.99	101.03	114.02	101.84	114.83	-	[34]
Benzene	1-Propanol	318.15	9.57	77.77	87.34	77.73	87.29	-	[35]
2-Butanone	2-Methoxyethanol	343.15	14.49	96.46	110.95	94.58	109.08	-	[35]

**Table 5:** Parameters of correlation activity coefficient and solubility calculation deviation.

Solute	*T*	*N*_p_	Parameters ^a^			A.A.D. (%)	A.R.D. (%)
	K		*C*	*D*	*E*		
Hydrogen	273.15	8	-	61.41	-	2.95	−0.74
Hydrogen	298.15	8	-	89.05	-	2.48	−0.87
Hydrogen	323.15	8	-	99.9	-	3.08	−1.53
Hydrogen	373.15	6	-	96.67	-	4.16	−2.77
Nitrogen	298.15	8	-	151.86	-	2.69	−0.06
Nitrogen	323.15	8	-	272.26	-	4.58	−0.13
Nitrogen	348.15	8		293.08		2.76	2.63
Nitrogen	373.15	7	-	177.98	-	6.66	4.50
Carbon dioxide	313.15	7	−4771.16	170.63	−1.58	0.51	0.01
Carbon dioxide	323.15	8	−3851.95	122.85	−1.02	1.35	0.01
Carbon dioxide	344.15	7	−3217.53	83.57	−0.57	3.64	0.08
Methane	324.65	6	−20,808.65	58.9	−0.02	0.26	0.00
Methane	375.65	6	−10,593.63	−17.44	−0.16	0.90	0.00
Methane	398.15	6	−2050.41	−46.15	0.08	0.66	0.01
Average						2.68	0.08

^a^ For hydrogen and nitrogen systems, $\ln\gamma_{1}=Dx_{1}$; for carbon dioxide and methane systems, $\ln\gamma_{1}=Cx_{1}^{2}+Dx_{1}+F$.

## Data Availability

All data generated or analyzed during this study are available within the article or upon request from the corresponding author.

## Supplementary Materials

The following supporting information can be downloaded at: https://www.mdpi.com/article/10.3390/ijms232112998/s1. Table S1. Calculated results of the proposed model [46,48,49,50,51].

## Nomenclature

A, B,C,D,E,a,b,c,d	the model constants for the model
$f_{1}^{L}$	fugacity of component 1 in the liquid phase [kPa]
$H_{i}$	Henry’s constant of solute *i* in the solvent [kPa]
$h^{E}$	excess enthalpy [kJ]
$\Delta h_{i}^{E,\infty }$	infinitely dilute molar enthalpy [kJ]
$N_{p}$	the number of experimental points
$n_{1}$	the molar composition of component 1 [mol]
p	total pressure [kPa]
$p^{s}$	saturated vapor pressure [kPa]
R	universal gas constant [J·mol^−1^·K^−1^]
$\Delta s_{i}^{E,\infty }$	infinitely dilute molar entropy [kJ·mol^−1^·K^−1^]
T	temperature [K]
V	volume of a system [m^3^]
$V^{E}$	excess volume [m^3^]
v	partial molar volume [cm^3^·mol^−1^]
${\bar{v}}_{1}$	partial molar volume of component 1 [cm^3^·mol^−1^]
$x_{i}$	liquid phase mole fraction of component *i*
$\gamma^{\infty }$	the activity coefficient of component *i* at infinite dilution
