# Supplementary material for: Research of a Thermodynamic Function (/)T, x→0: Temperature Dependence and Relation to Properties at Infinite Dilution
Source: Int J Mol Sci. 2022 Oct 27;23(21):12998. doi: 10.3390/ijms232112998 (PMC9656998; doi:10.3390/ijms232112998)
Supplement: Supplementary file 1 [file ijms-23-12998-s001.zip › ijms-1939004-supplementary.pdf]

Supplementary Material

Research of a thermodynamic function $(\frac{\partial p}{\partial x})_{T,x\rightarrow 0}$: temperature dependence and relation to properties at infinite dilution

Jiahuan Zheng[1], Xia Chen[2], Yan Wang[3], Qichao Sun[1], Wenting Sun[1], Lianying Wu[1,*], Yangdong Hu[1], Weitao Zhang[1]

[1] College of Chemistry and Chemical Engineering, Ocean University of China, Qingdao 266100, China[2] Institute of Chemical Engineering Guangdong Academy of Science, Guangzhou 510665, China

[3] College of Chemistry and Chemical Engineering, Qingdao University, Qingdao 266071, China

* Correspondence: wulianying@ouc.edu.cn

Table S1. Calculated results of the proposed model

Solute	T	H_1	p	$x_1{}^a \times 10^4$				AAD% [b]			Ref
	K	MPa	MPa	Exp.	This work 1[c]	This work 2[d]	K-K equation	This work 1[c]	This work 2[d]	K-K equation	
Hydrogen	273.15	5682.55	2.53	4.31	4.28	4.39	4.43	0.60	2.06	2.88	[46, 48]
			5.07	8.64	8.20	8.65	8.80	5.00	0.18	1.90	
			10.13	17.09	15.18	16.86	17.37	11.19	1.36	1.65	
			20.27	33.51	26.24	32.24	33.96	21.68	3.79	1.36	
			40.53	63.90	40.92	60.58	65.20	35.97	5.20	2.04	
			60.80	92.52	50.23	88.66	94.17	45.70	4.17	1.79	
			81.06	119.10	57.18	118.81	121.08	51.99	0.24	1.67	
			101.33	142.50	63.30	151.86	146.03	55.58	6.57	2.48	
Hydrogen	298.15	7005.30	2.53	3.50	3.42	3.53	3.60	2.28	0.81	2.81	[46, 48]
			5.07	6.96	6.54	6.96	7.16	5.97	0.04	2.88	
			10.13	13.86	12.05	13.64	14.18	13.03	1.61	2.31	
			20.27	27.20	20.62	26.27	27.86	24.18	3.40	2.44	
			40.53	52.50	31.35	50.03	53.99	40.29	4.70	2.84	
			60.80	76.40	37.32	73.69	78.64	51.15	3.55	2.93	
			81.06	99.10	40.95	98.96	101.84	58.68	0.14	2.77	
			101.33	120.60	43.51	127.34	123.78	63.92	5.59	2.64	
Hydrogen	323.15	7748.24	2.53	3.27	3.13	3.24	3.26	4.08	0.90	0.29	[46, 48]
			5.07	6.49	5.99	6.39	6.48	7.74	1.56	0.16	
			10.13	12.93	11.03	12.55	12.86	14.67	2.91	0.55	
			20.27	25.36	18.85	24.28	25.32	25.68	4.26	0.16	
			40.53	49.29	28.55	46.71	49.24	42.08	5.23	0.10	
			60.80	71.92	33.81	69.35	71.93	52.99	3.58	0.01	
			81.06	93.37	36.81	93.54	93.38	60.58	0.18	0.02	
			101.33	114.40	38.69	121.31	113.73	66.18	6.04	0.58	
Hydrogen	373.15	7220.53	2.53	3.71	3.37	3.49	3.49	9.01	5.69	5.73	[46, 48]
			5.07	7.32	6.47	6.94	6.95	11.58	5.11	4.97	
			10.13	14.48	11.99	13.80	13.78	17.16	4.72	4.86	
			20.27	28.38	20.78	27.33	27.06	26.80	3.69	4.65	
			40.53	54.66	31.71	53.78	52.37	42.00	1.61	4.19	
			60.80	79.61	38.41	82.93	76.00	51.75	4.17	4.54	
Nitrogen	298.15	8514.14	2.53	2.80	2.77	2.89	2.87	1.19	3.10	2.43	[46, 48]
			5.07	5.42	5.07	5.51	5.46	6.44	1.59	0.69	
			10.13	10.15	8.97	10.47	10.33	11.62	3.11	1.80	
			20.27	18.12	13.36	17.59	18.37	26.27	2.91	1.37	
			30.40	24.55	15.89	23.07	25.05	35.26	6.01	2.03	
			50.66	35.58	20.43	35.08	36.23	42.57	1.41	1.82	
			81.06	49.09	23.14	48.77	50.10	52.86	0.65	2.05	
			101.33	57.20	24.65	58.76	58.09	56.90	2.73	1.56	
Nitrogen	323.15	10926.61	2.53	2.19	2.14	2.27	2.25	2.25	3.76	2.68	[46, 48]
			5.07	4.36	3.96	4.46	4.37	9.25	2.19	0.34	
			10.13	8.12	6.80	8.48	8.26	16.30	4.42	1.71	
			20.27	14.70	9.20	13.73	14.99	37.42	6.62	1.95	
			30.40	20.34	10.26	17.85	20.73	49.55	12.23	1.90	
			50.66	29.82	13.48	30.35	30.49	54.81	1.78	2.25	
			81.06	41.81	13.60	42.46	42.62	67.47	1.55	1.95	

			101.33	49.00	13.44	51.01	49.65	72.58	4.10	1.33	
Nitrogen	348.15	11722.36	2.53	2.04	2.00	2.12	2.11	2.09	3.94	3.37	
			5.07	3.97	3.62	4.07	4.04	8.83	2.42	1.86	
			10.13	7.60	6.38	7.98	7.90	16.00	4.96	4.01	
			20.27	13.91	9.70	14.58	14.60	30.29	4.80	4.96	
			30.40	19.37	11.37	20.06	20.44	41.29	3.57	5.52	[46, 48]
			50.66	28.73	12.49	28.99	30.46	56.53	0.89	6.02	
			81.06	40.54	12.29	40.32	42.81	69.68	0.53	5.61	
			101.33	47.50	11.92	47.97	49.89	74.90	0.99	5.02	
Nitrogen	373.15	10104.35	5.07	4.15	4.44	4.78	4.86	7.01	15.21	17.20	
			10.13	7.89	7.66	8.81	9.11	2.96	11.67	15.44	
			20.27	14.67	12.19	15.82	16.94	16.93	7.86	15.48	
			30.40	20.56	14.88	21.45	23.74	27.65	4.32	15.46	[46, 48]
			50.66	30.64	17.63	30.41	35.39	42.46	0.74	15.50	
			81.06	42.95	19.26	41.36	49.60	55.17	3.71	15.47	
			101.33	49.81	19.88	48.25	57.52	60.09	3.14	15.48	
Carbon dioxide	313.15	233.95	5.07	162.00	173.06	162.37	162.82	6.83	0.23	0.51	
			7.60	205.00	221.85	204.19	202.34	8.22	0.39	1.30	
			10.13	220.00	249.87	219.16	220.87	13.58	0.38	0.40	
			15.20	232.00	279.85	232.54	232.42	20.62	0.23	0.18	[49]
			20.27	243.00	311.00	242.98	242.60	27.98	0.01	0.17	
			40.53	275.00	455.69	278.71	276.54	65.70	1.35	0.56	
			50.66	289.00	536.99	286.14	287.30	85.81	0.99	0.59	
Carbon dioxide	323.15	282.86	5.07	138.00	144.77	136.92	136.82	4.91	0.78	0.86	
			7.60	179.00	192.46	182.57	176.72	7.52	2.00	1.27	
			10.13	203.00	223.11	199.66	198.84	9.90	1.65	2.05	
			15.20	212.00	251.54	217.73	211.52	18.65	2.71	0.23	
			20.27	230.00	281.27	223.55	223.06	22.29	2.80	3.02	[49]
			30.40	247.00	344.26	246.71	242.82	39.38	0.12	1.69	
			40.53	262.00	409.93	263.21	257.07	56.46	0.46	1.88	
			70.93	301.00	678.23	301.81	298.28	125.32	0.27	0.90	
Carbon dioxide	344.15	386.00	10.13	166.00	183.39	171.59	163.67	10.47	3.37	1.41	
			12.67	182.00	205.08	183.37	177.83	12.68	0.75	2.29	
			15.20	197.00	226.35	191.06	190.68	14.90	3.01	3.21	
			20.27	213.00	257.55	201.21	204.65	20.92	5.53	3.92	[50]
			50.66	256.00	449.71	263.02	251.94	75.67	2.74	1.59	
			75.99	285.00	672.38	302.47	280.71	135.92	6.13	1.50	
			101.33	317.00	963.14	304.59	298.52	203.83	3.91	5.83	
Methane	324.65	5469.32	10.13	14.27	13.98	14.28	13.98	2.03	0.07	2.04	
			20.27	22.79	22.66	22.79	22.44	0.58	0.02	1.54	
			30.40	28.70	29.24	28.57	28.43	1.87	0.44	0.94	
			40.53	33.40	35.45	33.52	33.09	6.14	0.37	0.92	[51]
			50.66	37.30	40.96	37.43	36.88	9.82	0.34	1.13	
			60.80	40.90	46.33	40.78	39.96	13.27	0.31	2.29	
Methane	375.65	4991.64	10.13	13.55	16.54	13.52	16.25	22.09	0.22	19.92	
			20.27	22.05	28.46	22.20	27.19	29.08	0.66	23.32	
			30.40	28.70	38.24	28.44	35.22	33.22	0.91	22.72	[51]
			40.53	33.30	47.08	33.70	41.49	41.38	1.21	24.59	
			50.66	38.50	55.57	37.89	46.52	44.34	1.58	20.82	

			60.80	41.90	64.16	42.25	50.69	53.14	0.84	20.99	
Methane	398.15	5743.70	10.13	14.34	14.20	14.34	14.21	0.96	0.02	0.93	[51]
			20.27	23.21	24.09	23.19	23.80	3.77	0.09	2.52	
			30.40	29.60	31.80	29.52	30.68	7.45	0.25	3.65	
			40.53	34.30	38.45	34.72	35.86	12.11	1.21	4.54	
			50.66	39.60	44.58	38.96	39.81	12.58	1.61	0.53	
			60.80	43.00	50.65	43.33	42.97	17.80	0.77	0.06	
Average								32.41	2.68	4.27	

[a] The solubility of solute in water.

[b] $\text{AAD\%} = \frac{|x_{1,cal.} - x_{1,exp.}|}{x_{1,exp.}} \times 100\%$

[c] Calculated from Eq. (21)

[d] Calculated from Eq. (22)
